# Value of PCA3 to Predict Biopsy Outcome and Its Potential Role in Selecting Patients for Multiparametric MRI

**DOI:** 10.3390/ijms140611347

**Published:** 2013-05-28

**Authors:** Gisele H. J. M. Leyten, Elisabeth A. Wierenga, J. P. Michiel Sedelaar, Inge M. van Oort, Jurgen J. Futterer, Jelle O. Barentsz, Jack A. Schalken, Peter F. A. Mulders

**Affiliations:** 1 Department of Urology, Radboud University Nijmegen Medical Centre, P.O. Box 9101, 6500 HB Nijmegen, The Netherlands; E-Mails: g.leyten@uro.umcn.nl (G.H.J.M.L.); lieskes@hotmail.com (E.A.W.); m.sedelaar@uro.umcn.nl (J.P.M.S.); i.vanoort@uro.umcn.nl (I.M.O.); p.mulders@uro.umcn.nl (P.F.A.M.); 2 Department of Radiology, Radboud University Nijmegen Medical Centre, P.O. Box 9101, 6500 HB Nijmegen, The Netherlands; E-Mails: j.futterer@rad.umcn.nl (J.J.F.); j.barentsz@rad.umcn.nl (J.O.B.)

**Keywords:** prostate cancer, PCA3, MRI

## Abstract

PCA3 (prostate cancer gene 3) and multiparametric 3 tesla MRI are new promising diagnostic tools in the detection of PCa. Our aim was to study the clinical value of the Progensa PCA3-test: its predictive value for biopsy outcome, Gleason score and MRI outcome. We evaluated, retrospectively, 591 patients who underwent a Progensa PCA3-test at the Radboud University Nijmegen Medical Centre between May 2006 and December 2009. Prostate biopsies were performed in 290 patients; a multiparametric 3 tesla MRI of the prostate was performed in 163/591 patients. The PCA3-score was correlated to biopsy results and MRI outcome. The results show that PCA3 was highly predictive for biopsy outcome (*p* < 0.001); there was no correlation with the Gleason score upon biopsy (*p* = 0.194). The PCA3-score of patients with a suspicious region for PCa on MRI was significantly higher (*p* < 0.001) than in patients with no suspicious region on MRI (52 *vs.* 21). In conclusion, PCA3 is a valuable diagnostic biomarker for PCa; it did not correlate with the Gleason score. Furthermore, multiparametric MRI outcome was significantly correlated with the PCA3-score. Thus, PCA3 could be used to select patients that require MRI. However, in patients with a negative PCA3 and high clinical suspicion of PCa, a multiparametric MRI should also be done.

## 1. Introduction

Prostate cancer (PCa) is the second most commonly diagnosed cancer in men, accounting for 14% of all cancers in males [1]. Since the routine use of serum prostate-specific antigen (PSA) testing, PCa detection has increased considerably. In 2011, an estimated 903,500 men will be diagnosed with PCa worldwide, compared to 679,000 men in 2002 [1,2]. However, one of the main drawbacks of PSA testing is the lack of specificity, resulting in a high negative biopsy rate of 60%–75% [3]. Novel diagnostic approaches are required to improve our ability to detect PCa and to identify it better, for which patients require (repeat) prostate biopsy. PCA3 (prostate cancer gene 3) and 3 tesla multiparametric magnetic resonance imaging (MRI) are new promising diagnostic tools in the detection of PCa.

PCA3 is currently the most specific PCa gene. Since its identification in 1999, extensive research has been performed to assess the clinical utility of PCA3 in the diagnosis of PCa [4]. The current indication to use the PCA3-test is to aid in the decision to perform repeat biopsies. A cutoff value of 35 is used, as this gives an optimal balance between sensitivity (47%–69%) and specificity (72%–79%) to predict PCa in repeat biopsies [5–8]. In contrast, the specificity of a PSA level between 4.0 and 10.0 ng/mL to predict PCa in repeat biopsies is 25%–40% [3]. A recent study provided evidence that the PCA3-test could also be useful to aid in the decision to perform initial biopsies [9]. They suggested that a cutoff value of 20 might be optimal for men with no previous biopsies and a PSA level of 2.5–10.0 ng/mL. This would avoid 40% of biopsies; 95% of men with a Gleason ≥7 PCa had a PCA3-score ≥20.

Concurrently, multiparametric MRI is being used increasingly in the diagnostic process of PCa. This MRI examination consists of anatomic (T2W) images, dynamic contrast-enhanced (DCE) MRI, diffusion weighted imaging (DWI) and proton MR spectroscopic imaging (MRSI). These techniques have a high accuracy (80%–90%) to detect PCa in the gland [10–13], if read by an experienced radiologist. A recent study showed promising results for discrimination among different aggressiveness classes [14]. However, due to additional expenses and limited availability compared to the conventional transrectal ultrasound (TRUS)-guided biopsy, the routine application of MR-guided biopsy is not yet feasible. An additional, cheaper and more practical test to select patients who require multiparametric MRI and, potentially, subsequent MR-guided biopsy would be valuable.

Our aim was to study the value of the clinical use of the Progensa PCA3-test. We evaluated PCA3 for its predictive value for biopsy outcome, Gleason score and multiparametric MRI outcome. To our knowledge, the potential correlation between PCA3 and MRI outcome has never been evaluated before and is important in the light of the increasing role of MRI in the diagnosis of PCa.

## 2. Results and Discussion

### 2.1. The Value of PCA3 to Predict Biopsy Outcome

In total, 591 patients that had a Progensa PCA3-test were evaluated in this study. Patients’ characteristics and biopsy results are shown in Table 1. The indication for a Progensa PCA3 test was an elevated PSA level. Prostate biopsies were performed within six months after the PCA3-test in 290/591 patients. PCa was found in 41% of these biopsies. PCA3 was highly predictive for biopsy outcome, both when using PCA3 cutoff value 35 (*p* < 0.001) and 20 (*p* < 0.001). PCA3 was elevated (≥35) in 222/591 patients, of which 162 patients underwent biopsies. In total, 89/162 patients were diagnosed with PCa, leading to a positive predictive value (PPV) of the PCA3-test of 55%. Of the 369 patients with a PCA3-score <35, 128 underwent biopsies, of which 30 patients (23%) were diagnosed with PCa.

PSA and PCA3 characteristics are documented in Table 2, sorted by biopsy outcome. The median PCA3-score was higher in patients with a Gleason score ≥7 upon biopsy, compared to a Gleason score ≤6, but this difference was not statistically significant (68 *vs.* 56; *p* = 0.194). Almost all patients (92%) with a Gleason score ≥7 upon biopsy had a PCA3-score ≥20.

### 2.2. The Value of PCA3 to Predict Multiparametric MRI Outcome

**Figure 1:**
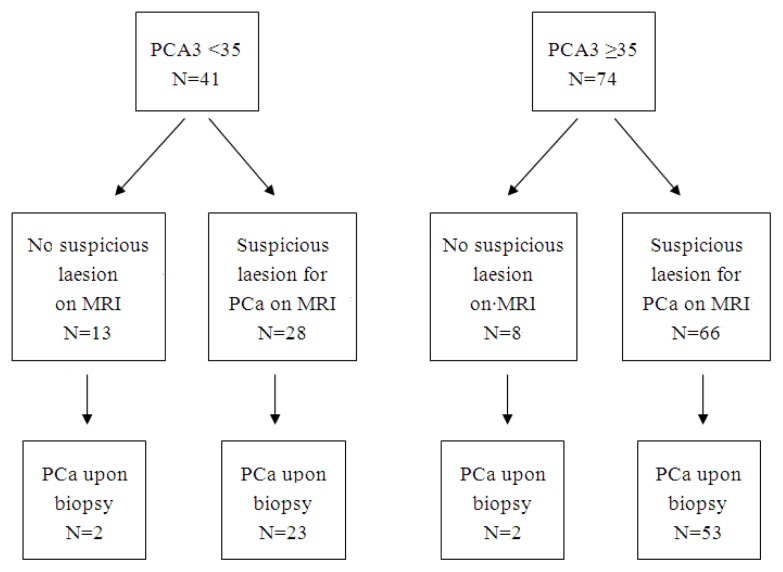
Flow diagram of the 115 patients that had a PCA3 test, multiparametric MRI and prostate biopsies.

A total of 115/290 patients that had a PCA3-test and prostate biopsies also underwent a multiparametric 3 tesla MRI of the prostate within 12 months (median, one month). The median follow-up was 24 months. The indication for performing MRI was set on clinical grounds, which had not been specifically documented. Patients with a suspicious region for PCa on MRI had a significantly higher PCA3-score than patients with no suspicious region (median 29 *vs.* 54, *p* = 0.002). The PSA level was not significantly different per MRI outcome (median 9.7 *vs.* 9.5, *p* = 0.650). Figure 1 shows a flow diagram with PCA3 score, MRI outcome and biopsy outcome. The majority of patients with PCA3 scores ≥35 had a suspicious region for prostate cancer upon MRI and were diagnosed with prostate cancer upon biopsy. In patients with PCA3 scores <35 and prostate cancer upon biopsy, almost all (23/25 patients) had a suspicious region for prostate cancer upon MRI. Only two patients with PCA3 scores <35 (PCA3 scores 2 and 7) and no suspicious lesion upon MRI were diagnosed with prostate cancer upon biopsy.

### 2.3. Discussion

In this study, the PCA3-score was significantly higher in patients with a suspicious region for PCa on MRI compared to patients with no suspicious region. To our knowledge, this is the first report to show a correlation between PCA3 and MRI outcome. There is one previous study in which PCA3 and MRI are combined [15]. However, they evaluated whether the potential value of the PCA3 test as a biomarker for prostate cancer diagnosis could be improved by the use of multiparametric MRI for redirecting (TRUS-guided) prostate biopsy. They did not correlate PCA3 score to MRI outcome; therefore, we cannot compare our results to the results of Sciarra *et al.* Our findings are particularly important in light of the increasing role of MRI in the diagnosis of PCa. Furthermore, we show that PCA3 was highly predictive for biopsy outcome, but was not significantly correlated with Gleason score upon biopsy.

Our study failed to show a correlation between PCA3 and Gleason score upon biopsy. The PCA3 score was somewhat higher in patients with a Gleason score ≥7 PCa compared to patients with a Gleason score ≤6 PCa (68 *vs.* 56), but the difference was not significant (*p* = 0.194). Recently, Auprich and colleagues concluded that the PCA3 score is low in indolent cancers, but that within the group of significant cancers, this marker did not further differentiate [16].

Several studies have been performed to evaluate the potential prognostic value of PCA3. Some studies documented a correlation of PCA3 with the Gleason score [7,9,17]. However, a range of studies reported no (additional) value of PCA3 to predict aggressive disease [16,18–20]. Our results are in agreement with Auprich *et al.*, *i.e.*, that the prognostic value of PCA3 is limited to discriminate indolent from significant cancer. Due to the low number (*n* = 10) of cases with indolent cancer in our study (according to the Epstein criteria: stage T1c, Gleason score ≤6, PSA density ≤0.15 and ≤33% positive cores upon biopsy), we cannot unequivocally support Auprich.

Even though PCA3 is a continuous variable, a PCA3 cutoff value of 35 is often used. However, the use of a PCA3 cutoff value is dependent on the indication. The study that led to FDA approval was based on a high negative protective value (NPV) (missing less prostate cancers and still preventing a considerable amount of prostate biopsies), which was achieved at a cut off of 20 (NPV 88%). In our study, the diagnostic value of PCA3 for predicting biopsy outcome was comparable when using a cutoff value of 20 or 35. However, of patients with a Gleason score ≥7 upon biopsy, 25% had a PCA3 score <35 and only 8% had a PCA3 score <20. These results correspond well to the results of de la Taille *et al.* [9], contributing to the evidence that a PCA3 cutoff value of 20 might be preferable.

**Figure 2:**
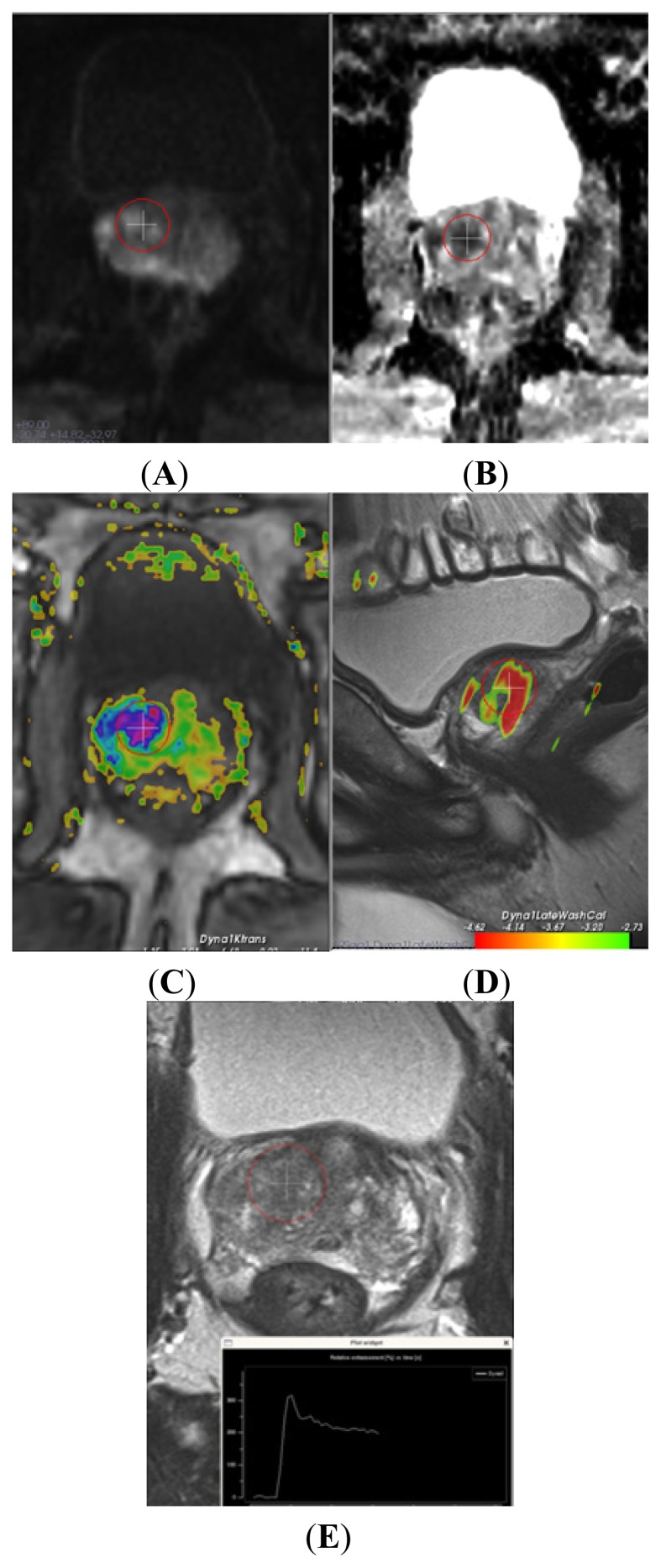
Multiparametric magnetic resonance (MR) imaging of the prostate of a 77 year old male, PSA level of 53, prostate cancer gene 3 (PCA3) score of 43 and 2× negative transrectal ultrasound-guided biopsy sessions. (**A**) axial diffusion weight imaging (DWI-b 1400) shows a high signal; this combined with a low signal on the (**B**) axial apparent diffusion coefficient (ADC) map, indicating restriction and, thus, suspicion for intermediate grade cancer. (**C**) axial and (**D**) sagittal dynamic contrast-enhanced MR images show increased vascular permeability. The curve (bottom) has a steep rise and wash out, which indicates cancer; (**E**) axial T2 weighted MR image shows a homogeneous low signal, which also fits tumor. MR-guided biopsy revealed in all three cores 75% Gleason 4+3 prostate cancer.

Multiparametric 3 tesla MRI is effective to detect and localize clinically significant PCa (an example is shown in Figure 2) [21,22]. In this retrospective study, we analyzed a subcohort of patients that underwent PCA3, multiparametric MRI and prostate biopsies. We show that PCA3 score is correlated with MRI outcome. The majority of patients with an elevated PCA3 score had a suspicious lesion upon MRI and was diagnosed with prostate cancer upon biopsies. Thus, PCA3, in addition to biochemical and clinical parameters, could be used to select patients that require MRI. However, due to the selection bias in this retrospective, observational study, our study is purely hypothesis generating and the clinical applicability of PCA3 to select patients that require MRI must be evaluated in prospective studies for its sensitivity, specificity, PPV and NPV. Also, the PCA3 cutoff value that will give an optimal balance between sensitivity and specificity to predict MRI outcome should be evaluated prospectively. In this study, using a PCA3 with a cutoff score of 20 did not change our results significantly (data not shown). Furthermore, we show that MRI might be of additional value in the subgroup of patients with a low PCA3 score and a high clinical suspicion of prostate cancer, as 23/28 patients with low PCA3 scores and a suspicious lesion upon MRI were diagnosed with prostate cancer. The NPV of the combined use of PCA3 and MRI was very high, as only two patients with low PCA3 scores and no suspicious lesion upon MRI were diagnosed with prostate cancer (Gleason of 6 and 8).

By demonstrating a correlation between PCA3 and MRI outcome, our study is the first step in the assessment of the clinical applicability of the combined use of PCA3 and MRI in the diagnostic process of prostate cancer. Based on our results, we hereby propose a prospective study design to further assess the hypothesis that PCA3 could select patients that require MRI. Ideally, all men in the prospective study would undergo a PCA3-test, multiparametric MRI and prostate biopsies. This would, however, lead to practical and financial objections. Therefore, we propose a prospective study in which men scheduled for TRUS-guided biopsies and a PSA <10 ng/mL will be randomized based on their PCA3-score. Men with a PCA3-score <20 will undergo TRUS-guided biopsies; men with a PCA3-score ≥20 will undergo MRI and subsequent biopsies if suspicious lesion(s) are seen on the MRI. We suggest a PCA3 cutoff score of 20, as this has been shown to detect the majority of men with clinically significant PCa.

## 3. Experimental Section

### 3.1. Study Design

All 591 patients who underwent a Progensa PCA3-test in the Urology outpatient clinic of the Radboud University Nijmegen Medical Centre between May 2006 and December 2009 were evaluated retrospectively. Urine samples were collected and Progensa PCA3-tests performed, as described by Groskopf *et al.* [6]. The indication to perform a PCA3 test was an elevated PSA level, a family history of PCa or previous negative prostate biopsies. Patient characteristics, number of previous biopsies, biopsy results, MRI outcome and follow-up results were documented. Prostate biopsies were performed within six months after the PCA3-test in 290 patients, using a 10 core biopsy regimen. Prostate biopsies were performed based on clinical grounds: PSA level, DRE (digital rectal examination) findings, family history and PCA3 score. A 3 tesla multiparametric MRI of the prostate was performed in 115/290 patients that also underwent prostate biopsies (40%).

MRI was performed using a 3 tesla MR scanner (Siemens Trio^®^ Tim). The multiparametric MRI consisted of a combination of anatomical T2-weighted T2W images with the following functional imaging modalities: DWI, DCE MRI and MRSI [21,22]. MRI was read by radiologists experienced in multiparametric MRI. Based on their MRI reports, MRI outcomes were categorized into two groups: no suspicious region for PCa or an evident suspicious region for PCa.

### 3.2. Statistical Analysis

The predictive value of PCA3 for biopsy outcome, Gleason score and MRI outcome was studied. Marker values were log-transformed to obtain a normal distribution. The independent sample *t*-test and the non-parametric Mann-Whitney U test were used to assess significance levels. Two-sided *p*-values of 0.05 or less were considered to indicate statistical significance. Statistical analyses were performed using Statistical Package for Social Sciences (SPSS, Chicago, IL, USA) version 16.0 for Windows.

## 4. Conclusions

PCA3 is a valuable diagnostic biomarker for prostate cancer, it did not correlate with biopsy Gleason score. Furthermore, there was a significant correlation of PCA3 with multiparametric MRI outcome. This generates the hypothesis that PCA3 could be used to select patients that require MRI. However, in patients with a negative PCA3 and high clinical suspicion of PCa, also a multiparametric MRI should be done. Based on our results, we propose a prospective study design to evaluate the clinical applicability of this hypothesis.

## Figures and Tables

**Table 1:** Patient characteristics and biopsy results.

	Total cohort (*n* = 591)		Biopsies taken * after PCA3 (*n* = 290)	No biopsy taken after PCA3 (*n* = 301)	*p*-Value
Age (median, yrs)	64		65	63	0.0230 a
DRE suspicious	117		90	27	<0.0001 b
Prostate volume (median, cc)	45		49	40	0.0267 a
No previous biopsies	255		167	88	<0.0001 b
PSA (median, ng/mL)	7.2		7.5	7.1	0.0432 a
PCA3 (median)	24		41	17	<0.0001 a
	PCA3 < 35 (*n* = 369)	PCA3 ≥ 35 (*n* = 222)			
Biopsy taken *	128	162			
Prostate cancer upon biopsy	30	89			

* Biopsy taken within six months after the PCA3 test; DRE, digital rectal examination; PSA, prostate-specific antigen.

a = Mann Whitney test;

b = Fisher exact test.

**Table 2:** PSA and PCA3 characteristics of patients who underwent biopsies within six months after the PCA3-test, sorted by biopsy outcome.

	No prostate cancer in biopsies (*n* = 148)	HG-PIN in biopsies (*n* = 23)	Gleason ≤6 prostate cancer in biopsies (*n* = 67)	Gleason ≥7 prostate cancer in biopsies (*n* = 52)
Age median (yrs)	63	65	65	67
PSA median (ng/mL)	6.6	6.3	7.9	14.1
PCA3 median	24	34	56	68
PCA3 ≥ 20	58%	78%	85%	92%
PCA3 ≥ 35	42%	48%	75%	75%

PSA, prostate-specific antigen; PCA3, prostate cancer gene 3; HG-PIN, high-grade prostatic intraepithelial neoplasia.
